# Fault Diagnosing of Cycloidal Gear Reducer Using Statistical Features of Vibration Signal and Multifractal Spectra

**DOI:** 10.3390/s23031645

**Published:** 2023-02-02

**Authors:** Iwona Komorska, Krzysztof Olejarczyk, Andrzej Puchalski, Marcin Wikło, Zbigniew Wołczyński

**Affiliations:** Faculty of Mechanical Engineering/Kazimierz Pulaski University of Technology and Humanities in Radom, 26-600 Radom, Poland

**Keywords:** cycloidal gearbox, diagnostics, multifractal analysis

## Abstract

The article presents a method for diagnosing cycloidal gear damage on a laboratory stand. The damage was simulated by removing the sliding sleeves from two adjacent external pins of the cycloidal gearbox. Damage to the sliding sleeves may occur under operating conditions and can lead to the destruction of the gear unit. Hence, early detection is essential. Signals from torque sensors, rotational speed sensors and vibration acceleration sensors of input and output shafts for various rotational speeds and transmission loads were recorded. The frequency analysis of these signals was carried out. Due to the fluctuation of the rotational speed, the frequency spectrum gives an approximate picture and is not useful in detecting this type of damage. The statistical characteristics of the signal were determined. However, only statistical moments of higher orders, such as kurtosis, are sensitive to the tested damage. Therefore, the use of multifractal analysis of the vibration signal using the wavelet leader method (WLMF) was considered. Then log-cumulants of the multifractal spectrum were selected as the new signal features.

## 1. Introduction

In robotics, high-precision gears, mostly cycloidal, are used to achieve the required high accuracy. Cycloidal drives [1] are used in around 75% of the joints of industrial robots. The second most commonly used gearbox is Harmonic Drive with approximately 20% of utilization in the wrist of industrial robots, while in collaborative robots or robots with small payloads, the harmonic drive is located in all joints. Presented in [2] is a review of the gearboxes used in robotics, along with a proposed robotic transmission assessment framework that includes the most crucial gearbox properties such as control, safety, weight and compactness, efficiency and productivity. The authors of cycloidal drive research primarily focus on the influence of theoretical calculations [3,4,5], design [6,7,8], manufacturing [9,10] and assembly parameters on the dynamics behaviour of the cycloidal gearbox, including contact [11]. One of the key factors, the efficiency of the industrial gearboxes [12,13,14,15,16,17], is also extensively researched.

The different failure modes of the cycloid reducers can present themselves as: gear tooth fracture, bearing wear, pitting, or even cracks on sleeves, etc. Modes are usually dependent on each other and can be considered when assessing gearbox reliability, including time-variant reliability analysis of industrial robots equipped with the RV cycloidal gearbox using the Kriging model [18]. (RV is the two-stage reducer that connects cycloidal and involute gears).

The cycloidal gearbox represents a special case of the reducer due to the principle of speed reduction; the vibration analysis can determine some fundamental train frequencies, along with mesh frequencies, while some of the operating frequencies are difficult to define. Nevertheless, an approach to predictive maintenance was undertaken in [19], which focused on the cycloidal ring gear housing, disc and eccentric bearing fault diagnostics. The principles of cycloidal box working cause torque fluctuation, which is observed on the output of the gear [20]. This phenomenon, along with the machining tolerances [21], affect the backlash of the gearbox and the amplitude spectrum [22], which can additionally affect the fault diagnostics.

The fault detection on the cycloid RV reducer is based on the vibration signals, with the incorporation of two traditional artificial intelligence methods: artificial neural network and support vector machine, into the noise convolution network model (NOSCNN) [23]. The method was used to determine different failure modes of the RV speed reducer (planetary gear and cycloidal wheel fault, as well as a needle defect) for varying working conditions, different rotating speeds and loads connected with additional noise. This research [24] proposes damage detection of the cycloidal gearbox utilizing the vibration signal. The vibrations are collected for cyclo-stationary and non-cyclo-stationary working conditions of the bench simulating the robot’s arm movement. Papers [25,26] describe the method based on condition indicators of the envelope spectrum of the vibration signal. The author presents bearing fault detection for a cycloidal gearbox during a run-to-failure test. In [27], a model of the vibration signal of the cycloidal drive was developed for diagnostic purposes, considering the internal loads distribution. The model is intended to be a diagnostic tool capable of simulating various damages resulting from the natural wear of components.

The most popular method of diagnosing gear damage is the analysis of signals recorded from vibration velocity or vibration acceleration sensors. Particularly easy to use are accelerometers that allow the obtaining of information without disassembling the machine over a wide frequency range. In the case of stationary signals, an effective analysis method is the frequency spectrum [28]. However, in most cases, the rotational speed fluctuates, leading to a loss of frequency spectrum resolution. Then, it would be necessary to perform an initial resampling of the signal [29] or use other methods that improve the resolution, such as coherence methods [30]. Diagnosis is often carried out in real time and, to avoid preprocessing, time-frequency methods are used to analyze dynamic signals. In [31], the authors review the time-frequency analysis used in condition monitoring. Particularly noteworthy is wavelet analysis [32,33]. The Vold-Kalman filtering (VKF)-based compound faults diagnosis method, based on time-frequency analysis, was presented in [34] in its application to gears and bearings of rotating machines with variable rotational speed. Another approach is based on a reference model [35,36] called the autoregression model, determined by the signal averaging technique. A symptom of damage is a residual signal and its features. Synchronous methods, such as the so-called order-tracking analysis [37,38], are also applicable. They allow synchronization of the signal with the shaft rotation and eliminate the influence of noise on the measurements. For non-stationary signal analysis, the empirical mode decomposition (EMD) algorithm can be used, which decomposes the signal into empirical components [39]. A noteworthy approach to diagnosing damage and wear of gears is presented by the authors, e.g., in [34,40,41,42], treating the vibration signal as a second-order cyclo-stationary process and demonstrating the effectiveness of this method.

In recent years, automated diagnostic procedures have been developed more often, eliminating the need for a qualified diagnostician. In the era of Industry 4.0, a large amount of data from measuring sensors is collected and processed by data mining algorithms. Paper [43] reviews various diagnostics techniques that have been shown to be successful when applied to rotating machinery. It also highlights fault detection and identification techniques based mainly on vibration analysis approaches. A short description of a new approach to diagnosis using fuzzy neural networks, is included.

A relatively new trend in diagnosing mechanical damage is the fractal theory. It is useful when analyzing non-stationary and non-linear signals. The most commonly used method is detrended fluctuation analysis (MF-DFA). Papers [44,45,46] describe its application for diagnosing damage to rolling bearings and, in [47,48,49], for detecting errors in toothed gears. An adaptive version of this method is presented in [50]. Unfortunately, it has disadvantages and the main one is the sensitivity to the selection of analysis parameters. The algorithm of wavelet leaders multifractal formalism (WLMF) does not have this disadvantage. Image analysis is presented in [51] as an example of application. The WLMF method’s use in diagnosing rotating machines is described in [52].

The paper presents a method of diagnosing damage to the sliding sleeves of the external pins of a cycloidal reducer. It should be emphasized that in the literature it is difficult to find articles on diagnosing damage to cycloidal gears. This publication partially fills this gap. The results of the vibration and torque analysis are presented. In addition, an innovative diagnostic method combining data-driven analysis and fractal theory was used. It is based on the method of wavelet leaders (WLMF). This method was used to diagnose multiple faults in a conventional gear transmission on a laboratory stand [53]. Damage symptoms are defined as multifractality level, span of dimensions and singularity with the greatest dimension. The presented work used a modified version of the method to detect damage to the cycloidal gear. New signal features were defined as log-cumulants. This method is used for the first time to diagnose damage to the cycloidal gear.

The main highlights of the work are:

(1) Analysis of the vibration and torque signal of a cycloidal gear reducer for different speeds and loads for the gear in fault-free condition as a function of time and frequency.
(2) Analysis of changes in the time course and frequency spectrum under the conditions of the tested damage: the sliding sleeves of external pins.
(3) The use of the wavelet leaders multifractal analysis (WLMF) algorithm to determine the multifractal spectrum and log-cumulants as characteristics of the vibration signal enabling the automation of damage diagnosis.

The manuscript is structured as follows: after the introduction in Section 1, Section 2 presents the reducer test bench and the necessary mathematical concepts. Then, experimental results are presented in Section 3, while in Section 4 the algorithm from Section 2.3 is applied to the measured data. Finally, in Section 5, conclusions are presented.

## 2. Materials and Methods

### 2.1. Cycloidal Gearbox

**Figure 1:**
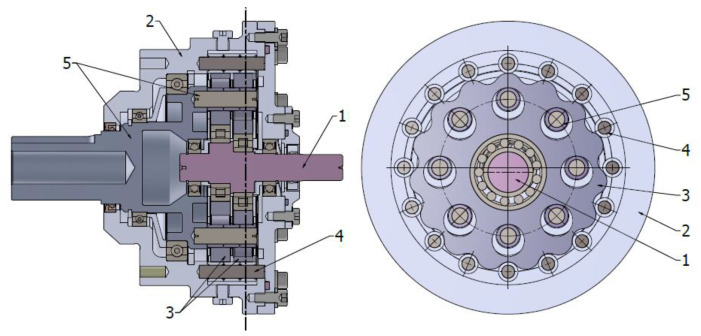
Cycloidal gear: 1—input shaft, 2—housing, 3—cycloidal discs, 4—outer pins with sliding sleeves, 5—output shaft with inner pins and sliding sleeves.

The cycloidal gear Figure 1 was designed as a gear with the internal tooth profile and with a ring gear design as part of a housing [15]. The main components of the gearbox were made of steel e.g., cycloidal discs, shafts and external and internal pins, while sliding sleeves mounted on internal and external pins were made of sintered bronze. The speed reduction ratio for the gearbox is 15.

The cross-sectional view of the investigated cycloidal gearbox is shown in Figure 1. The gearbox consists of two cycloidal discs (3) with epi-cycloidal teeth placed on the input shaft eccentrics (1). The cycloidal discs during rotation are shifted in phase from each other at 180 degrees. The discs mesh with external pins assembled into the ring gear (4), which is integrated into the housing (2). The torque from the input shaft (1) to the output shaft (5) is transferred via a output flange consisting of internal pins mounted in the output shaft (5). The inner pins are in the holes of two cycloidal discs (3). The larger diameter of the holes of the cycloidal disc allows the rotational motion of the pins. The number of cycloidal disc teeth equals the reduction ratio of the gearbox i = 15, while the number of external pins is one higher. The number of internal pins is equal to 8. The dimensions of the pins and their amount strictly depend on the assumed maximal input torque and reduction ratio [8].

### 2.2. Experimental Stand

**Figure 2:**
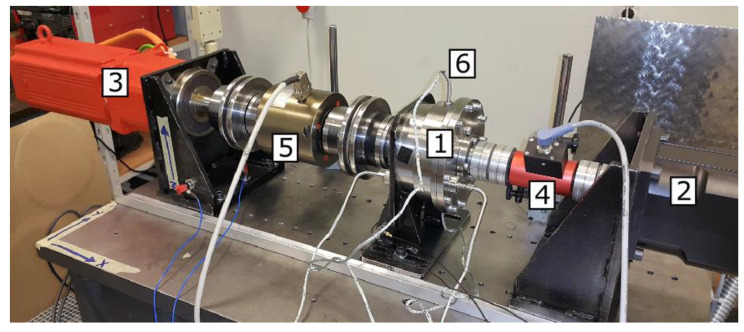
Test bench setup: 1—tested cycloidal gear, 2—driving electric motor, 3—braking electric engine, 4—torque and velocity meter for input, 5—torque and velocity meter for output.

The test stand (Figure 2) was set up in such a way that it was possible to work in different working modes with varying speeds and torques with feedback from torque meters.

To achieve a higher degree of flexibility of the bench, the input DC electric motor (2) was chosen with a rated input speed of 6000 rpm (maximal 9000 rpm) and a torque of 6 Nm. The braking unit (3) is a DC electric motor capable of working with torque up to 180 Nm. Two KTR DataFlex torque sensors equipped with additional speed indicators with 10 Nm and 200 Nm on the input (4) and output (5) of the gearbox, respectively, were used to collect the torque measurements. The input torque was measured using DataFlex 16/10 (Nominal torque −10–+10 Nm, Output Voltage Torque −10–+10 V, No of impulses/revolution 360, Error in linearity <0.1%), while the output torque-DataFlex 42/200 (Nominal torque −200–+200 Nm, Output Voltage Torque 0–10 V, No of impulses/revolution 60, Error in linearity <±0.5%). The components were connected using flexible connectors to avoid misalignment errors.

**Figure 3:**
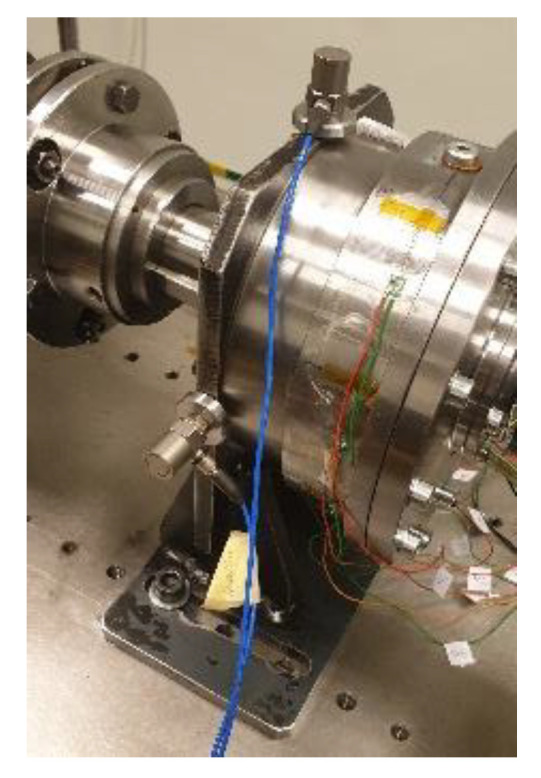
Placement and direction of installation of accelerometers.

For collecting a vibration signal, two monoaxial DeltaTron accelerometers, Bruel&Kjaer type 4395 (Frequency range 0.3–18,000 Hz, Sensitivity 1.0 mV/m/s^2^ ±0.2%), were utilized. The sensors were installed on the reducer’s support using magnetic holders (see Figure 3). The first sensor was installed in the horizontal direction and the second sensor was installed in the vertical. For synchronizing the collected data from accelerometers, torque and speed sensors, the bench was additionally equipped with a laser Monarch PLT200 Tachometer (RPM range 0.5–20,000, Accuracy Laser +/−0.01%/Contact +/−0.05%, Resolution 0.001 to 10 RPM) that gave information regarding the position of the input or output shaft of the gear. Additionally, the testing bench was equipped with thermocouples. Two of them were mounted inside the gear from the top and bottom sides through the threaded holes for oil fill-in and were used to control the lubricant temperature. The other two were utilized to monitor the gearbox housing temperature and the ambient one. All the measurements were performed at the same temperature during a whole measuring cycle. The data acquisition and control, were performed using the National Instrument hardware with LabView software. The tests were performed with the oil level determined for the highest efficiency, which was 55% of the total gear capacity. The tests with optimal oil level were performed to mimic the real working conditions at which the gears are supposed to be exploited and for which fault diagnostics should work. For test purposes, synthetic oil with API class GL-5 with a viscosity 75 W-90 was chosen [54].

**Figure 4:**
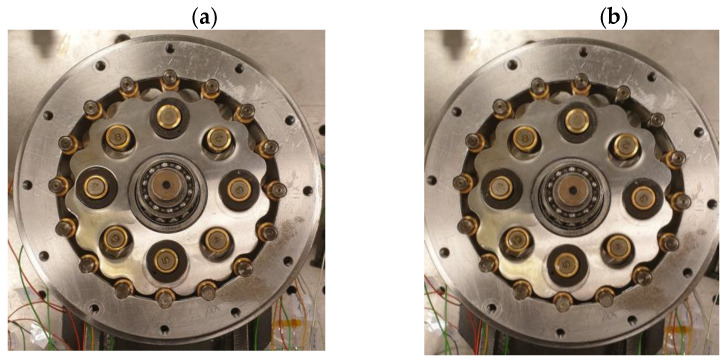
Cycloidal gear (**a**) complete (fault-free), (**b**) with the sliding sleeves removed on the two adjacent outer pins (faulty).

External pins are one of the most loaded parts in the gearbox due to contact pressure. The tested gear was equipped with sleeves made from sintered bronze, which broke during the fault. The simplest way to simulate fault with this kind of sleeve without destroying the testing gearbox is to remove them. Taking this into account, the damage was simulated by removing the sliding sleeves from two adjacent external pins of the cycloidal drive (Figure 4).

### 2.3. Multifractal Formalism

The multifractal formalism allows a statistical analysis of time series using Holder exponents. Holder’s exponents h of the time function $x\left(t\right)$ at time moment $t_{0}$ are determined by the supremum of exponents satisfying condition (1), for C=const>0, where $P_{n}\left(t-t_{0}\right)$ is a polynomial of the order n<h

(1) $$\left|x\left(t\right)-P_{n}\left(t-t_{0}\right)\right|\leq C{\left|t-t_{0}\right|}^{h},$$

The histogram of the estimated scaling exponents, referred to as the $D\left(h\right)$ multifractal spectrum, enables the study of the regularity of the time signal. The multifractal spectrum determines the fractal dimensions of singularity subsets with a given exponent value [55,56,57].

The signal scaling exponent estimation algorithm implemented in the time-frequency domain allows the estimation of multifractal parameters using wavelet leaders (WLMF), defined using the wavelet coefficient of the discrete wavelet transform (DWT) of the function $x\left(t\right)$. The algorithm with low computational costs and high stability works well for various real signals [51,52].

Coefficients $d_{x}\left(j,k\right)$ of the discrete wavelet transform (DWT) and the basic wavelet with a compact support $\psi_{0}\left(t\right)$ of the function $x\left(t\right)$, wavelet leaders $L_{x}\left(j,k\right)$ for the set of the largest coefficients $d_{x}\left(j^{\prime },k^{\prime }\right)\equiv d_{\lambda^{\prime }}$ in the interval of 3I on any scale are described with dependencies (2) and (3), respectively [53]

(2) $$d_{x}\left(j,k\right)=\int_{\mathbb{R}}^{}x\left(t\right)2^{-j}\psi_{0}\left(2^{-j}t-k\right)dt,$$

(3) $$L_{x}\left(j,k\right)=\underset{I^{\prime }\in 3I}{\sup}\left|d_{I^{\prime }}\right|,$$

where j,k are integers and $3\lambda ∶=3\lambda_{j,k}=\lambda_{j,k-1}\cup \lambda_{j,k}\cup \lambda_{j,k+1}$ and $\lambda ∶=\lambda_{j,k}=\left[k2^{j},\left(k+1\right)2^{j}\right]$. $L_{x}\left(j,k\right)$ consists of the largest wavelet coefficient $d_{x}\left(j^{\prime },k^{\prime }\right)$ computed at all finer scales $2^{j^{\prime }}\leq 2^{j}$ within a narrow time neighborhood $\left(k-1\right)\cdot 2^{j}\leq 2^{j^{\prime }}k^{\prime }<\left(k+2\right)\cdot 2^{j}$.

Holder exponents are the scaling exponents of wavelet leaders: $L_{x}\left(j,k\right)\sim 2^{jh}$. Moreover, the structure function (4) defined for wavelet leaders is described by the power dependence, the exponent of which is the multifractal scaling exponent $\zeta \left(q\right):\mathbb{R}\to \mathbb{R}$

(4) $$Z_{L}\left(q,j\right)=\frac{1}{n_{j}}\sum_{k=1}^{n_{j}}L_{x}{\left(j,k\right)}^{q}\sim F_{q}2^{j\zeta \left(q\right)},$$

where q is the order of the structure function and $n_{j}$ is the number of intervals of the multiresolution analysis.

The upper limit for the multifractal spectrum (5) of the tested signal can be obtained by the Legendre transformation of the multifractal scaling exponent $\zeta \left(q\right)$, under mild conditions of signal regularity [58]

(5) $$D\left(h\right)\leq \underset{q\neq 0}{\min}\left[1+qh-\zeta \left(q\right)\right],$$

where h is the Holder’s exponent and $D\left(h\right)$ the multifractal spectrum.

Decomposition of the scaling exponent $\zeta \left(q\right)$ (6) into a Taylor series allows estimation of multifractal spectrum parameters using the log-cumulant $c_{p}$ of the order p≥1 [53]

(6) $$\zeta \left(q\right)=\sum_{p=1}^{\infty }c_{p}\frac{q^{p}}{p!},$$

The location of the maxima of the multifractal spectrum is described by log-cumulant $c_{1}$, while the level of multifractality, i.e., the width of the spectrum and the asymmetry, are described by log-cumulants $c_{2}$ and $c_{3}$, respectively. If the number of zero moments of the wavelet is twice the largest Holder exponent of the signal, the scaling exponents of the structural function are independent of the choice of the wavelet.

## 3. Results

Measurements were made for three rotational speeds (1000, 2000 and 3000 rpm) and five loads (0, 18, 22, 26 and 30 Nm). Ten records of approximately 1 min were registered. The records were divided into time samples of 5 s, covering approximately 10–11 revolutions of the output shaft. This allowed for a frequency spectrum resolution of 0.2 Hz to be obtained.

The sensors were installed on the reducer’s support using magnetic holders, so the frequency range was limited. The frequency range of 3 kHz was enough for further analysis. An appropriate anti-aliasing filter was also used.

**Figure 5:**
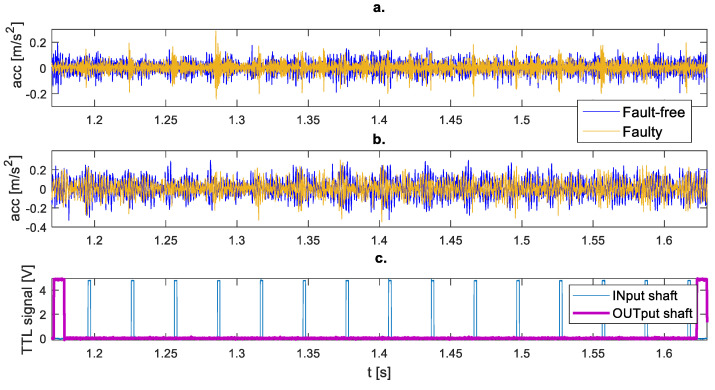
Vibration acceleration waveform during 1 revolution of the output shaft at a speed of 2000 rpm and a load of 30 Nm: (**a**) Vertical vibrations, (**b**) Horizontal vibrations, (**c**) Tachometers signals.

Figure 5 shows an example of waveforms of vibration acceleration signals in the vertical (a) and horizontal (b) directions for fault-free and simulated fault conditions during one revolution of the output shaft. Indicators of rotation of the input (INput shaft) and output (OUTput shaft) shafts are marked in Figure 5c. The simulated damage causes the appearance of impulse responses in the vibration signal, especially in the vertical direction (Figure 5a), although the signal amplitude values change slightly.

**Figure 6:**
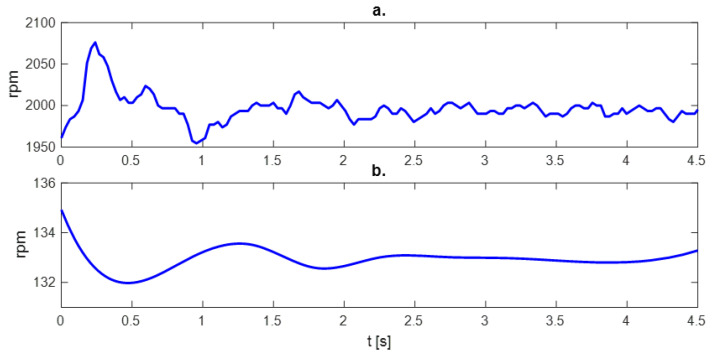
Fluctuation of (**a**) input and (**b**) output shaft rotational speed.

It is worth noting that the rotational speed of the shafts during gear operation is floating, even at a fixed rotational speed, which can be seen in Figure 6.

**Figure 7:**
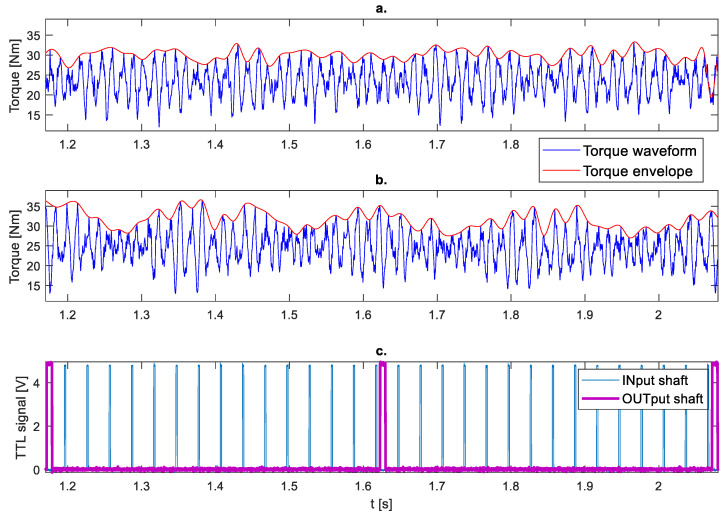
Torque waveform during two revolutions of the output shaft at a speed of 2000 rpm and a load of 30 Nm: (**a**) Fault-free, (**b**) Faulty, (**c**) Tachometers signals.

Figure 7 presents the results of measurements of the instantaneous torque of the output shaft during two revolutions of the output shaft for fault-free and faulty conditions with signal envelope. The envelope amplitude of torque signals for fault-free and faulty states was determined. In the first case (Figure 7a), the amplitude of the envelope is 15.3 Nm, while after damage (Figure 7b) is 25.9 Nm. It can be seen that, in the faulty state, the amplitude of the signal envelope increases. Indicators of rotation of the input (INput shaft) and output (OUTput shaft) shafts are marked in Figure 7c.

## 4. Further Analysis

Further analysis of the vibration signal included signal resampling and frequency analysis of the vibration acceleration signal and torque.

**Figure 8:**
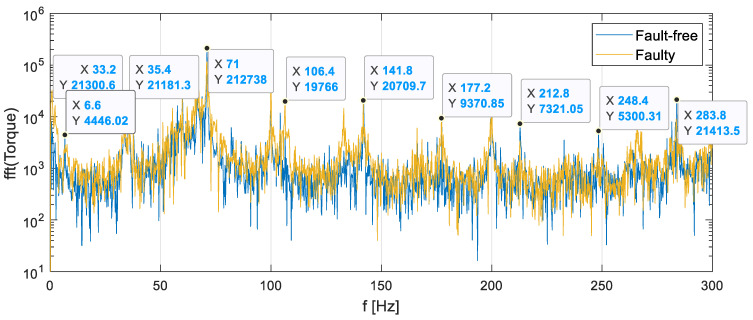
Frequency spectrum of the torque signal of the output shaft at a speed of 2000 rpm and a load of 30 Nm.

Figure 8 compares the averaged frequency spectra of the torque signal measured on the gearbox output shaft for fault-free and faulty conditions at a rotational speed of 2000 rpm and a load of 30 Nm.

The main frequency components of the spectrum of the torque fluctuations and vibrations are listed in Table 1.

A conventional input shaft with 15 teeth would have a gear mesh frequency of rpm x 15. However, the cycloid gear has a relative motion to the shaft, driven by the eccentric gear and the output shaft. The relative motion of the cycloid to the ring gear means that, for each revolution, there is one extra gear mesh [25].

All spectrum components listed in the table have their multiples in the spectrum. Comparing the frequency spectra of the torque signal in Figure 7, it is difficult to notice significant differences between the signals registered for fault-free and faulty conditions.

**Figure 9:**
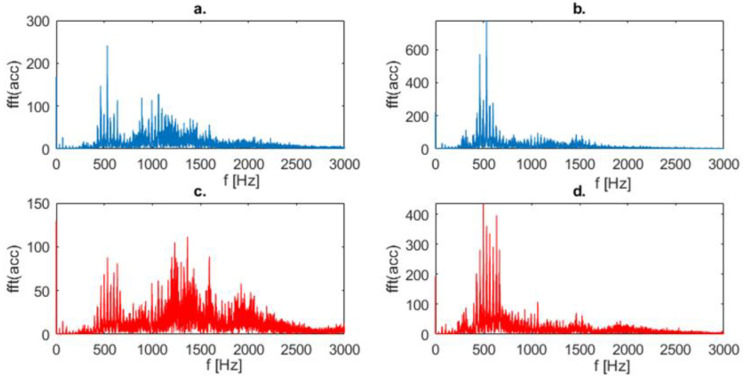
Frequency spectrum of vibration acceleration signal: (**a**) Vertical vibrations for fault-free state, (**b**) Horizontal vibrations for fault-free state, (**c**) Vertical vibrations for faulty state, (**d**) Horizontal vibrations for faulty state.

A similar analysis was performed for the vibration acceleration signal. Its results are shown in Figure 9. A qualitative difference was noted for vertical vibrations (compare Figure 9a,c).

**Figure 10:**
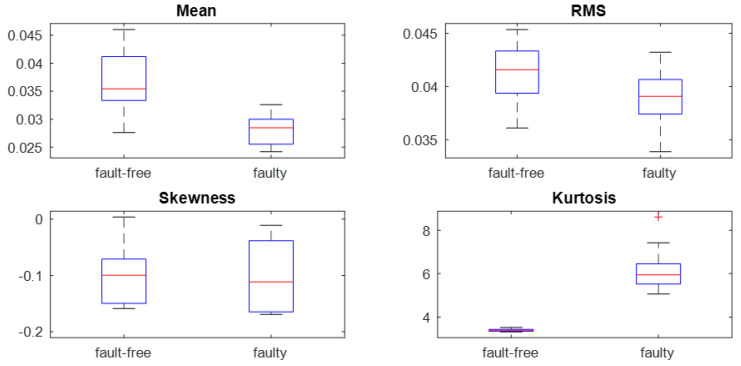
Statistical features of the vibration signal for fault-free and faulty conditions at rotational speed of 2000 rpm and load of 30 Nm.

Statistical features of the signal were determined: the mean value, RMS, skewness and kurtosis of the vibration signals (Figure 10). For each state, approximately 120 values of each statistical feature were obtained, which were used to determine the box plots.

Apart from kurtosis, all statistical moments show a similar value for both fault-free and faulty conditions. Higher values of kurtosis testify to the impulsive nature of the vibration response to damage.

**Figure 11:**
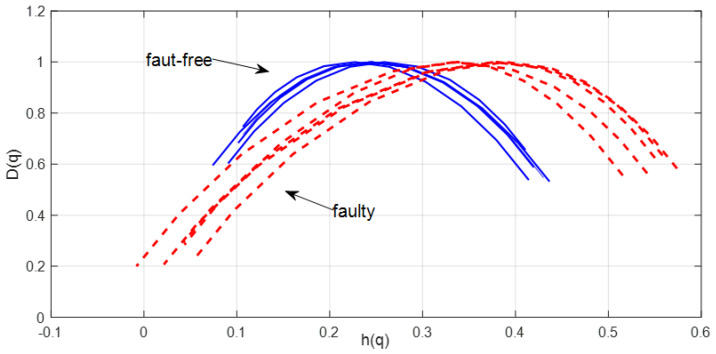
Multifractal spectra for fault-free (blue line) and faulty conditions (red dashed line).

Due to the appearing impulse responses in the time signal of vibrations in the vertical direction, a multifractal analysis of the signal by the wavelet leader method was selected. Multifractal spectra were determined for time windows covering about two revolutions of the output shaft. Figure 11 shows multifractal spectra for fault-free (blue line) and faulty conditions (red dashed line).

Multifractal spectra for the damaged transmission show a higher level of multifractality, i.e., a wider spectrum and a shift of the spectrum peak to the right at the tested damage.

**Figure 12:**
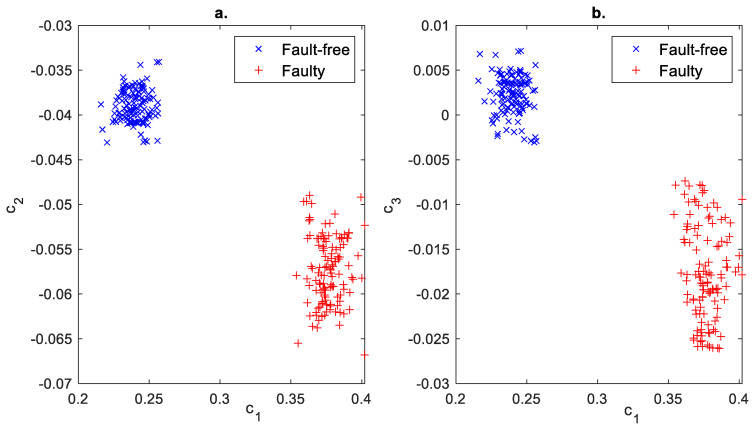
Fault classification using log-cumulants of the multifractal spectrum using (**a**) $c_{1}-c_{2}$ plane, (**b**) $c_{1}-c_{3}$ plane.

The cumulants of the multifractal spectrum were used for damage detection and are presented in Figure 12.

Based on the values in Figure 12, it can be concluded that, using the cumulants of the multifractal spectrum, it is possible to fully distinguish the fault-free state from the faulty state. The faulty condition is characterized by a greater spread of cumulative values than the fault-free state. Two cumulants, e.g., $c_{1}$ and $c_{2}$ or $c_{1}$ and $c_{3}$, would suffice for a single failure test. The cumulants $c_{2}$ and $c_{3}$ are characterized by the largest dispersion of values than $c_{1}$. For the fault-free state, the cumulants are more concentrated than for the faulty state.

## 5. Conclusions

The article describes the detection method of the cycloidal gear damage that was simulated by removing the sliding sleeves from two adjacent external pins of the cycloidal gearbox. Damage to the sliding sleeves may occur under operating conditions and lead to the destruction of the gear unit. The frequency spectra and statistical features of the vibration acceleration signal were analysed. The vibration signal recorded in the vertical direction is advantageous.

The frequency spectrum is not of much use due to small differences in the amplitudes of the spectrum components characteristic of the gears. The use of the frequency spectrum is also burdensome due to fluctuations in the rotational speed of the transmission and requires preliminary resampling of the vibration signal.

The sensitivity of the basic statistical features of the vibration acceleration signal was tested. Average values, RMS values and skewness are of no use in fault detection; only kurtosis gives a good discrimination result. However, literature research and experience with other toothed gears show that the impulsive nature of vibration waveforms is caused by most gear damage and, thus, increases the value of kurtosis and statistical moments of higher orders.

The presented work describes a method of automatic data-driven diagnostics based on log-cumulants of the multifractal spectrum for the examined damages. The algorithm at this stage of research is entirely satisfactory. The shape and position of the multifractal spectrum are sensitive to small changes in the nature of the vibration signal. The measures of this change are logarithmic cumulants. In this way, three new diagnostic features are introduced that can be useful in the multi-fault classification of cycloidal gear reducers.

In the future, it is planned to investigate other damage to the cycloidal gear reducer, such as bearing wear or damages, gear tooth fracture, pitting, etc. Then, comparative tests of different methods will be performed for multi-fault defects detection.

## Figures and Tables

**Table 1:** The main frequency components of the spectrum of the torque fluctuations and vibrations signals.

The Spectrum Component	Frequency [Hz]	Frequency at 2000 rpm [Hz]
Speed frequency of input shaft	rpm of input shaft/60	33.3
Speed frequency of output shaft (modulation)	rpm of output shaft/60 or rpm of input shaft/*i*/60	2.22
Mesh frequency	rpm/*i*·(*n*_o_ + 1)	35.56
Mesh frequency harmonics	*k*·rpm/*i*·(*n*_o_ + 1); *k* = 1,2,3…	71.1, 106.67, 142.23, …

where *i* is gear ratio, *n*_o_ number of gear teeth.

## Data Availability

Not applicable.
